# Demographic and anatomic predictors of glenoid morphology: a systematic review

**DOI:** 10.1016/j.xrrt.2026.100676

**Published:** 2026-01-29

**Authors:** Ravi Prakash, Andrea L. Aagesen, Nashra Javed, Elizabeth L. Yanik, Nitin B. Jain

**Affiliations:** a Department of Physical Medicine and Rehabilitation, University of Michigan, Ann Arbor, MI, USA; b Department of Orthopaedic Surgery, University of Michigan, Ann Arbor, MI, USA; c Department of Physical Medicine and Rehabilitation, University of California, Los Angeles, CA, USA; d Department of Orthopaedic Surgery, Washington University School of Medicine, St. Louis, MO, USA; e Department of Orthopaedic Surgery, The University of Texas Southwestern Medical Center, Dallas, TX, USA

**Keywords:** Glenoid morphology, Glenoid wear patterns, B2 glenoid, Glenohumeral osteoarthritis, Glenohumeral joint, Total shoulder arthroplasty

## Abstract

**Background:**

The glenohumeral (GH) joint displays diverse glenoid morphology or wear patterns that affect joint alignment, stability, and function. These morphological variations are influenced by sex, ethnicity, and height, potentially limiting the effectiveness of standardized prosthetics. While previous studies have linked glenoid shape to shoulder instability and bone loss, the demographic and anatomic predictors for glenoid morphology or specific glenoid wear patterns remain unclear. This review aims to identify key demographic and anatomical predictors of glenoid morphology to inform surgical planning and improve total shoulder arthroplasty outcomes.

**Methods:**

We performed a comprehensive literature search of MEDLINE/PubMed, EMBASE, and Web of Science with a search cutoff date of August 2024. We included the studies that addressed demographic and anatomic predictors, such as age, sex, height, hand dominance, laterality, and glenoid morphology. A methodological quality assessment was conducted independently as per standardized set of criteria known as Newcastle–Ottawa Quality Assessment Scale.

**Results:**

We retrieved a total of nine studies that reported on demographic and anatomic predictors and glenoid morphology. Seven of these studies were of good quality, while two were of fair quality. Glenoid size was consistently larger in males and positively correlated with age, height, and weight. We also observed that subtype B2 glenoid is linked to increased humeral osteophyte length in primary GH osteoarthritis.

**Conclusion:**

Age, male sex, and height are associated with increased glenoid dimensions and version, while subtype B2 glenoid correlates with advanced GH osteoarthritis and bone loss. B2 glenoid present greater surgical challenges requiring individualized approaches. Larger studies are needed to confirm these predictors and explore genetic and molecular links to disease severity.

Total shoulder arthroplasty (TSA) is an effective procedure to treat primary glenohumeral (GH) osteoarthritis (OA) for alleviation of pain and restoration of shoulder function.^15^ Despite the high success rate of TSA, the number of revision arthroplasties is steadily increasing.^9^ One of the most common complications following TSA is implant loosening, particularly involving the glenoid side, often leading to the need for revision surgery.^5^ This highlights the critical importance of precise positioning of the implant components for long-term success in TSA.^10^

The GH joint exhibits a spectrum of glenoid wear patterns^28^^,^^29^^,^^40^ that significantly influence joint alignment and stability, thereby affecting a wide range of shoulder motions/functions.^37^ There is considerable variation in glenoid morphology, not only between individuals but also across different ethnic groups.^21^^,^^28^^,^^29^^,^^40^ These variations can affect the suitability of standardized prosthetic components, supporting the need for tailored preoperative planning. Customized prosthetics or surgical techniques may be necessary to accommodate these diverse morphologies.

Walch originally classified glenoid morphology in primary GH OA into three main categories with five subtypes.^37^ More recently, the modified Walch classification introduced new subtype B3, type D glenoid, and a more precise definition of the A2 glenoid.^3^ This modification aims to enhance interobserver and intraobserver reliability in classifying the complex patterns of glenoid deformity.^3^ The B2 glenoid (biconcave) is often associated with posterior head subluxation, leading to posterior glenoid bone loss. Studies have shown that B1 has a higher rate of progression to B2 or B3 subtypes compared to A1 glenoid, indicating a higher risk of progression to severe glenoid deformity over time.^38^^,^^43^ Accurate glenoid morphology classification can help identify individuals at risk for developing severe glenoid deformities, allowing for better management and treatment planning.

A few studies have reported ethnicity, sex, and height as predictors of glenoid shape and size, but the role of these demographic and anatomic predictors in predicting glenoid wear patterns remains unclear. Earlier studies have focused on glenoid morphology as predictor for anterior and posterior GH joint instability or dislocations and glenoid bone loss.^1^^,^^11^ Other studies have evaluated glenoid erosion patterns with B2 glenoid in patients undergoing TSA.^7^^,^^19^^,^^27^ To date, there has not been a comprehensive review of the literature to assess the potential demographic and anatomic predictors of variable glenoid wear patterns.

The present systematic review was conducted to comprehensively assess the available literature to identify and evaluate evidence regarding the potential demographic and anatomic predictors associated with glenoid wear patterns. We hypothesize that age, ethnicity, and sex are prominently associated with glenoid wear patterns. Understanding these relationships will help inform more effective surgical planning and improve TSA outcomes.

## Methods

### Search strategy and study selection

We conducted a systematic review as per Preferred Reporting Items for Systematic Reviews and Meta-Analyses guidelines.^26^ A protocol was developed before conducting the literature search and was registered in International Prospective Register of Systematic Reviews (CRD: 42023446234).^6^ A comprehensive literature search was performed across the following databases: MEDLINE (PubMed), EMBASE, and Scopus, with a search cutoff date of August 2024. A detailed description of keywords, medical subject headings, and title/abstract (tiab) terms used to identify the studies investigating associations between demographic and anatomic predictors and glenoid morphology can be found in Supplementary Table S1.

We exported the search results from each database into EndNote 20 bibliographic software (Thompson Reuters, New York, NY, USA). The references were then transferred into Covidence (http://www.covidence.org), an online platform that facilitated the initial screening of titles and abstracts according to the predefined eligibility criteria.^2^ Two independent researchers (RP and NJ) screened all the titles and abstracts based on the criteria described. In case of disagreement between the two reviewers, a third researcher (NBJ) made the final decision regarding the inclusion of articles. We included studies that reported demographic and anatomic predictors, including age, sex, body mass index (BMI), height, and hand dominance in relation to variability in glenoid morphology, including glenoid version, glenoid inclination, and patterns of glenoid wear. Eligible studies were limited to those with cross-sectional, case-control, or retrospective design based on prospectively collected data and were published in the English language. Studies were excluded if they were not original research (editorials, opinions, systematic reviews, and meta-analysis). Animal studies and biomarker studies were also excluded.

### Quality assessment

A methodological quality assessment was conducted for each article included by two independent reviewers (RP and NBJ), who evaluated the studies using Newcastle–Ottawa Quality Assessment Scale guidelines.^39^ Based on the Newcastle–Ottawa Quality Assessment Scale scoring criteria, the studies were classified into three categories: good (7-8 stars), fair (5-6 stars), and poor (4 stars).

### Assessment of demographic and anatomic predictors

We evaluated age, sex, height, weight, BMI, laterality, hand dominance, and shoulder pathology as possible demographic and anatomic predictors.

### Data abstraction

The full-text articles for the selected studies were retrieved, and data were abstracted in a spreadsheet. A standard approach was used to extract data from each article: study title, date of the publication, journal, first author, study design, glenoid morphology, glenoid wear patterns, age, sex, number of cases and controls, demographic and anatomic predictors, and association estimates (unadjusted effect estimate and multivariable adjusted effect estimates, if available). Studies with estimates for two or more independent populations (eg, men and women in different age groups) were also documented.

## Results

### Search results

**Figure 1:**
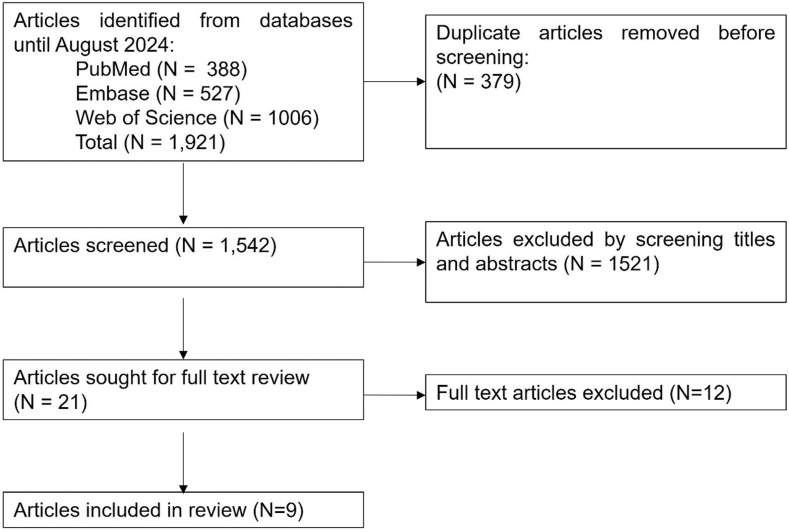
PRISMA flow diagram for the systematic review. *PRISMA*, Preferred Reporting Items for Systematic Reviews and Meta-Analyses.

Figure 1 illustrates the flowchart of inclusion of studies in this systematic review. We observed a total of 1,921 articles after a complete search, of which 379 articles were removed due to duplication of articles. Out of 1,542 articles, 1,521 were excluded after screening for title and abstract. Finally, twenty-one articles were retrieved for full-text review. Among those, 12 articles did not report on any demographic and anatomic predictors. Finally, nine articles reporting on demographic and anatomic predictors related to age, sex, height, weight, BMI, laterality, hand dominance, and shoulder pathology were included (Fig. 1).

### Study characteristics and quality assessment

We found nine studies with 4,521 patients, published between 2013 and 2024. All of the studies were cross-sectional originating from five different countries: 1 study from USA,^22^^,^^36^ four different studies from Turkey,^18^^,^^24^^,^^31^^,^^35^ 1 from Indonesia,^33^ two from Germany,^12^^,^^36^ and 1 from Japan.^42^ For assessment of glenoid parameters, 1 study used magnetic resonance imaging on a 1.5-T magnetic resonance imaging scanner,^12^ 1 used axillary radiographs alone,^22^ 1 used a combination of axillary radiographs and computed tomography (CT) imaging,^36^ three used CT scans alone,^18^^,^^31^^,^^35^ and three used 3D CT scans.^24^^,^^42^ The NOS was used by classifying eight studies of good quality, one studies were of fair quality (Tables I and II).

**Table I:** Characteristics of studies included.

Title	First author	Yr	Study design	Study size	Country	Mean age (range or ± SD)	Imaging modalities	Males: females	Other relevant details	Inter- and intra-rater reliability
Glenoid morphology and related parameters in Turkish society	Gokhan Karademir^18^	2022	Retrospective cross-sectional	102 shoulders from 51 patients	Turkey	41.69 yr (18-73)	CT imaging	27 males: 24 females	Determining the mean glenoid size and retroversion and examine association with sex, dominant side, height weight and BMI	Not reported
Axillary view: arthritic glenohumeral anatomy and changes after ream and run	Frederick A. Matsen III^22^	2013	Retrospective cross-sectional	344 shoulders	USA	-	Radiographs (axillary)	213 males: 127 females	Arthritic shoulders within 1 mo before a shoulder arthroplasty	High
Sex and side differences of 3D glenoid anthropometric parameters in a normal Turkish population	Abdulhamit Misir^24^	2019	Retrospective cross-sectional	200 shoulders	Turkey	56.3 ± 12.5 yr (18-66)	3D-CT	100 males: 100 females	Glenoid anthropometric parameters in Turkish population	High
Assessment of glenoid morphology in the Turkish population	Abdulkadir Sari^31^	2020	Retrospective cross-sectional	140 patients	Turkey	39.6 ± 11.8 yr (18-60)	CT imaging	78 males: 62 females	Evaluation of glenoid version, height and width based on age, sex, height, side and hand dominance in Turkish population	Moderate
Sex determination from glenoid cavity by CT in Turkish population	Mehmet Ulkir^35^	2022	Retrospective cross-sectional	200 scapulae	Turkey	18-93 yr	CT scans	100 males: 100 females	Sex-stratified length, breadth and depth of glenoid cavity	High
Is there a correlation between humeral osteoarthritis and glenoid morphology according to Walch?	P. Vetter^36^	2022	Retrospective cross-sectional	143 shoulders from 135 patients	Germany	69.3 yr (47-85)	Radiographs (axillary) and CT imaging	62 males: 73 females	Patients undergoing TSA for primary GH OA with an intact rotator cuff	High
Impact of age on glenoid size: three-dimensional CT analysis of glenoid anatomy	Kotaro Yamakado^42^	2023	Retrospective cross-sectional	380 patients	Japan	67.2 ± 10.1 yr (27-89)	3D-CT	229 males: 151 females	Predictors of glenoid size	Not reported
Morphology of humeral head and glenoid in normal shoulder of Indonesian population	Heri Suroto^33^	2022	Cross-sectional	71 patients	Indonesia	57.8 ± 9.9 yr (28-77)	CT scan	25 males: 46 females	Shoulder morphology from Indonesian population	Not reported
Gender-specific factors influencing the glenoid version and reference values for it	Cornelius Sebastian Fischer^12^	2024	Cross-sectional	3,004 patients	Germany	52.8 ± 13.8 yr (21-90)	MRI	1,443 males; 1,561 females	Gender specific factors influencing glenoid version	Low

*SD*, standard deviation; *CT*, computed tomography; *3D*, three-dimensional; *MRI*, magnetic resonance imaging; *TSA*, total shoulder arthroplasty; *BMI*, body mass index; *GH OA*, glenohumeral osteoarthritis.

**Table II:** Quality assessment of studies included in the systematic review as per Newcastle–Ottawa quality assessment scale.

Articles	Selection				Comparability	Outcome/exposure			Total score
	1	2	3	4	1	1	2	3	
Karademir and Aslan^18^	∗	∗	∗	∗	∗∗	∗	∗	-	8 (G)
Matsen III and Gupta^22^	∗	∗	∗	∗	∗∗	∗	∗	∗	9 (G)
Misir et al^24^	∗	∗	∗	-	∗∗	∗	-	∗	7 (G)
Sari et al^31^	∗	∗	∗	-	∗	∗	∗	-	6 (F)
Ulkir et al^35^	∗	∗	∗	-	∗	∗	∗	∗	7 (G)
Vetter et al^36^	∗	∗	∗	∗	∗	∗	-	∗	7 (G)
Yamakado^42^	∗	∗	∗	∗	∗∗	∗	∗	-	8 (G)
Fischer et al^11^	∗	∗	∗	∗	∗∗	∗	∗	-	8 (G)
Suroto et al^33^	∗	∗	∗	-	∗	∗	∗	∗	7 (G)

Stars: ∗ (1 star given), ∗∗ (2 stars given); quality ratings: G (good quality), F (fair quality); selection domain: 1 (adequate case definition), 2 (representativeness of cases), 3 (selection of controls), 4 (definition of controls); comparability domain: 1 (comparability of cases and controls based on study design or analysis); exposure domain: 1 (ascertainment of exposure), 2 (same method of ascertainment for cases and controls), 3 (nonresponse rate).

Of the studies, seven examined the relationship between gender and glenoid morphology, and size, while one study further examined the association with glenoid retroversion, humeral contact, and glenoid type as defined by the Walch classification.^18^^,^^22^^,^^24^^,^^31^^,^^33^^,^^35^^,^^42^ Two studies explored disease impact on glenoid morphology, specifically GH OA,^22^^,^^36^ two compared right and left shoulders,^24^^,^^31^ three studied hand dominance,^18^^,^^31^^,^^42^ two studied age,^31^^,^^42^ and five studied height, one of which included BMI and weight.^12^^,^^18^^,^^24^^,^^31^^,^^42^

## Study results

### Sex vs. glenoid size

Six of the eight included studies evaluated the association between sex and glenoid dimensions using measures such as anteroposterior (AP) diameter, superoinferior (SI) diameter, height, width, and surface area. All consistently demonstrated significantly larger glenoid dimensions in males when compared to females.^18^^,^^24^^,^^31^^,^^35^^,^^42^ One study reported significant right–left differences in addition to sex differences. The right glenoid was significantly larger in height (37.5 ± 3.3 mm vs. 36.2 ± 2.9 mm; *P* = .037) and width (26.1 ± 2.3 mm vs. 24.6 ± 1.4 mm; *P* = .005), suggesting a potential influence of shoulder dominance.^24^ Yamakado et al^42^ also demonstrated significantly greater difference for glenoid height and width in males and females. Similarly, a study form Indonesia reported significantly larger dimensions in males (height: 37.6 ± 2.1 mm; upper width: 20.0 ± 1.9 mm; and lower width: 27.2 ± 2.0 mm) compared with females (height: 32.6 ± 2.6 mm; upper width: 17.6 ± 1.9 mm; and lower width: 23.2 ± 2.0 mm)^33^ (Table III). Overall, the evidence consistently demonstrates larger glenoid size in males across all measured parameters.

**Table III:** Sex-stratified comparison of glenoid morphology.

Glenoid height, width, and version	Male	Female	*P* value	Reference
Glenoid height	28.74 ± 3.07 mm	22.48 ± 3.24 mm	<.01	Karademir and Aslan^18^
Glenoid width	31.3 ± 3.89 mm	26.58 ± .57 mm		
Glenoid version (degree)	−1 ± 0.89	−0.98 ± 0.97		
Glenoid height	39.1 ± 2.5 mm	34.5 ± 1.8 mm	–	Misir et al^24^
Glenoid width	27.7 ± 2.2 mm	23.6 ± 1.6 mm		
Glenoid version (degree)	−6.7 ± 3.4	−4.4 ± 3.7		
Glenoid height	41.1 mm	38.8 mm	<.0001	Yamakado et al^42^
Glenoid width	31.2 mm	26.1 mm		
Glenoid length	38.54 ± 2.61 mm	34.04 ± 2.21 mm	<.001	Ulkir et al^35^
Glenoid breadth	27.83 ± 2.69 mm	23.77 ± 2.14 mm		

### Sex vs. glenoid version

Five studies examined sex-related differences in glenoid version.^18^^,^^21^^,^^22^^,^^24^^,^^31^ There was a dichotomy in findings; two studies reported significant sex differences, while the remaining studies reported none. Three studies (two from Turkey and 1 from Indonesia) found no significant sex-based differences in glenoid retroversion.^18^^,^^31^^,^^33^ Sari et al^22^ found no significant differences in glenoid version between males and females in the Turkish population. Conversely, a study based on axillary radiographs identified significant sex differences, with females exhibiting a larger glenoid angle (*P* < .001). This study also reported a higher prevalence of type A glenoids in females (80%) and type B glenoids in males (53%) (*P* < .001). Another Turkish study reported significantly different glenoid version values between sexes (*P* = .037).^24^ A recent large study (n = 3,004) demonstrated that males tended to have retroverted glenoids (−1.5 ± 4.4 [−42.5 to 11.1]), whereas females demonstrated slightly anteverted glenoids (0.2 ± 3.6 [–17.7 to 12.7]).^12^ Thus, sex-related differences in glenoid version remain inconsistent across studies.

### Age vs. glenoid morphology

Two studies evaluated the relationship between age and glenoid morphology.^31^^,^^42^ A Turkish study (mean age, 39.6 ± 11.8 years) did not report any significant association between age and glenoid version.^31^ By contrast, a Japanese study (mean age 67.2 ± 10.1 years) identified a significant age-related increase in glenoid dimensions, with each additional year associated with a 0.043 mm increase in glenoid height and a 0.042 mm increase in width (*P* < .0001 for both).^42^ Fischer et al^12^ further reported a positive association between increasing age and anteverted glenoid version (*P* < .001). These findings indicate that aging is associated with enlargement of glenoid dimensions and in some cohorts, increased anteversion.

### Height vs. glenoid morphology

Four studies consistently reported a positive correlation patient height and glenoid dimensions.^18^^,^^24^^,^^31^^,^^42^ All observed significant increases in glenoid dimensions with increasing height (*P* < .05 or *P* < .01).^24^^,^^42^ Two studies also provided predictive models. A Turkish study demonstrated a 16.6 mm increase in height (*P* < .0001) and 14.1 mm increase in width (*P* < .0001) for every additional meter in height.^24^ Another study from Japan reported a linear regression model, height = 103.849 + 2.422 x glenoid width (*P* = .000), further quantifying the positive relationship between taller stature and larger glenoid size.^42^ In contrast, two studies evaluating height and glenoid version found no significant association, suggesting that height influences glenoid size but not angular orientation.^18^^,^^31^

### Body mass index and weight vs. glenoid morphology

One study found weight to be strongly correlated with glenoid dimensions (r = 0.70 for diameter; r = 0.66 for height; *P* < .01), but not with glenoid version (*P* = .81).^18^ BMI showed no significant correlation with glenoid dimensions or version (*P* value: .14 for diameter, .3 for height, .46 for glenoid version).^18^ Similarly, another recent study also did not show any association between BMI and glenoid version.^12^ These findings suggest weight primarily appears to be a proxy for height in its association with glenoid dimensions.

### Glenoid wear patterns vs. disease

Two studies examined the association between glenoid wear patterns and shoulder pathology.^21^^,^^36^ One study demonstrated a strong relationship between humeral head osteophyte length (OL) and GH OA severity and eccentric glenoid morphology.^36^ B2 and B3 glenoids were observed to be significantly associated with length of humerus osteophytes (<3 mm in grade I GH OA to 13 mm in grade IV GH OA; *P* < .0001). A humeral head OL of ≥13 mm was strongly indicative of eccentric glenoid types B2 and B3 (odds ratio, 14.20; 95% confidence interval [CI], 5.96-33.85). The study further noted an increase in glenoid retroversion with the OA severity of OA, with the mean angle rising from 3.1° in grade I to 17.3° in grade IV (*P* < .0001). This trend supports the association between humeral OL and glenoid retroversion (r = 0.707), as well as with posterior humeral subluxation (r = 0.452). The study identified humeral OL, posterior humeral subluxation, and glenoid retroversion as independent predictors for advanced glenoid morphology, with respective odds ratios of 1.17 (95% CI, 1.03-1.32; *P* = .013), 1.11 (95% CI, 1.01-1.22; *P* = .031), and 1.48 (95% CI, 1.30-1.68; *P* < .001). These findings underscore a strong interplay between degenerative disease progression and glenoid morphology.

### Glenoid morphology vs. laterality

Two Turkish studies reported contrasting findings regarding the effects of laterality on glenoid version and dimensions.^24^^,^^31^ For glenoid version, one study (good quality) found no significant differences between right and left shoulders (*P* > .05).^31^ A second study (fair quality) reported significant laterality-related variation with differences in glenoid retroversion between sides (*P* = .000).^24^ For glenoid size, the first study again reported no significant differences (*P* > .05 for AP and SI measurements).^31^ Conversely, the second study reported significant differences, with the right glenoid larger in height (*P* = .037) and width (*P* = .005).^24^ These conflicting results underscore variability in how laterality may influence glenoid morphology.

### Glenoid morphology vs. hand dominance

Two studies consistently found that hand dominance significantly affects glenoid version.^18^^,^^31^ Hand dominance was significantly associated with glenoid version; right-hand–dominant individuals had more retroverted glenoid on the dominant side, and similarly, left-hand–dominant individuals showed more retroversion on their dominant side.^31^ Both instances demonstrated statistical significance with *P* values <.01. The second study corroborated these findings, indicating significant retroversion on the dominant side with a *P* value <.01.^18^ Three other good quality studies investigating the effect of hand dominance on glenoid morphology have shown variable association.^18^^,^^31^^,^^42^ Two Turkish studies found no significant association between dominance and glenoid dimensions (*P* values >.05 for AP and SI).^18^^,^^31^ In contrast, a Japanese study found significantly larger glenoid size on the dominant side (*P* < .0001).^42^ Overall, hand dominance shows a consistent influence on glenoid version but inconsistent effects on glenoid size.

## Discussion

Our systematic review demonstrates several consistent patient-related factors associated with glenoid morphology. Across studies, males exhibited larger glenoid dimensions than females, and increasing age was associated with incremental enlargement of both glenoid height and width. Patient height also showed a uniform positive correlation with glenoid size, with two studies providing quantitative predictive models supporting this relationship. Weight similarly demonstrated a strong association with glenoid dimensions, although BMI did not. Disease severity contributed meaningfully to morphological variations, with B2 and B3 glenoids correlating with greater humeral OL, particularly when humeral head osteophytes exceeded 13 mm in primary GH OA. In addition, two studies reported that hand dominance significantly influenced glenoid version, with individuals consistently demonstrating increased retroversion on the dominant side, an observation aligning with prior findings that higher-demand or more active shoulders tend to exhibit greater retroversion.

The relationship between age and glenoid wear patterns is complex. Only one of the studies in our review supports that the increase in age is associated with the increase in glenoid size. Similar findings have been reported that older patients exhibited larger humeral heads and increased glenoid surface areas compared to their younger counterparts, even in the absence of radiographic signs of OA.^4^ This challenges the assumption that changes in bone size parameters inevitably signify radiological signs of OA, highlighting the influence of the physiological aging process. Moreover, the study suggests that the degenerative process during aging, coupled with the weakening of joint-stabilizing soft tissues such as the rotator cuff, may contribute to enlargements in the glenoid-to-head ratio. This enlargement could serve as an indicator of improved osseous stabilization in response to age-related degeneration and the weakening of supporting structures.

Our review shows that there is significant sexual dimorphism involved in glenoid size, with males typically exhibiting larger dimensions in various parameters, such as AP diameter, SI diameter, height, width, and surface area. These findings have crucial implications for the design and selection of prosthetics, emphasizing the need for sex-specific approaches. Specifically, the variability in glenoid cavity volume, breadth, length, depth, and perimeter is not only of anatomical interest but also clinically significant in determining the fit and sizing of glenoid components during shoulder replacement surgery. Therefore, to avoid complications developing after shoulder arthroplasties refer to soft tissue irritation, impingement, or instability of the implant and prosthesis design which must cater to the sexual dimorphism in glenoid dimensions.

While significant variations in glenoid dimensions have been reported across different ethnicities, it is important to note that the available data is largely limited to individuals of European ancestry and Japanese. The Japanese population had mean glenoid dimensions 28.1 ± 1.6 mm (width) and 35.8 ± 2.2 mm (height) in males, and 23.4 ± 1.7 mm (width) and 30.8 ± 1.8 mm (height) in females.^32^ One of the studies on French population reported mean glenoid dimensions of 28.7 ± 2.1 mm (width) and 37.3 ± 1.9 mm (height) in men, and 24.7 ± 1.7 mm (width) and 33.5 ± 1.8 mm (height) in women.^25^ Non-Hispanic White Americans were observed to have mean glenoid dimensions of 29 ± 3.1 mm (width) and 39 ± 3.7 mm (height).^16^ Despite evident differences in glenoid dimensions among genders and ethnicities, contemporary prostheses have predominantly targeted European and American patient populations. This emphasis may introduce a misalignment between existing glenoid designs and the actual dimensions of glenoid specimens. Mizuno et al^25^ noted this discrepancy, pointing out that the minimum baseplate size in prosthesis designs in 2017 was 25 mm, potentially inadequately accommodating smaller dimensions. This underscores the importance of addressing anthropometric diversity in prosthetic design to ensure optimal outcomes across various patient demographics.

Our review shows that glenoid dimension increases along with the increase in height, suggesting a statistically significant correlation with the size of glenoid cavity. This indicates a correlation where taller individuals will have larger glenoid cavities. In contrast, when the focus shifts to weight and BMI, the association with glenoid morphology becomes less clear. While weight alone shows a strong correlation with the size of the glenoid, BMI—a composite measure of weight and height—does not show a correlation with glenoid dimensions. The lack of significant correlation between BMI and both glenoid dimensions and version hints at the nuanced influence of body composition on bone morphology, suggesting that height may play a more critical role than weight or body fat percentage.

One of the key findings we observed is the link between humeral OA and eccentric glenoid morphology. This relationship is posited to result from humeral osteophyte formation leading to posterior humeral translation.^36^ This phenomenon is likely due to the decrease in joint space caused by formation of osteophytes. This leads to the deformation of both humeral head and glenoid and results in uneven contact surface in GH joint, further progressing GH OA. This contact, especially on the posterior aspect after osteophyte formation, is proposed to emerge as a significant starting point for the eventual cascade of glenoid bone erosion or loss.^38^ The process can be conceptualized as follows: humeral translation, driven by elevated OL, leads to contact with the posterior glenoid, marking the inception of a sequence that may culminate in glenoid bone erosion or loss.

Patients with OA demonstrated a less retroverted glenoid angle compared with those who had avascular necrosis or cuff tear arthropathy, and their humeral contact position was significantly more posterior.^36^ Larger humeral OL showed a strong association between eccentric glenoid types B2 and B3 and GH OA. Posterior glenoid wear pattern has been shown to produce greater bone loss than central wear seen in type A glenoids, which might be contributing to the larger humeral OL observed in type B glenoid wear patterns.^17^^,^^20^ Increasing glenoid retroversion parallels advancing GH OA severity, and studies consistently demonstrate that progressive humeral OL corresponds with more advanced and eccentric glenoid wear. Walker et al^38^ observed a trend of more frequent medialization of type B glenoid over time compared to type A glenoid, which contributes to the larger humeral OL associated with type B glenoid. Iannotti et al's^17^ findings of higher medial wear in type B3 glenoid than type B2 glenoid support the idea that type B3 glenoid may evolve from type B2 glenoid, potentially explaining the larger OL in type B3 glenoids. Notably, similarities in humeral OL between type A2 and type B1 glenoid indicates a transition, suggesting that type B1 glenoid might have inherited OL characteristics from type A1 glenoid. Moreover, the association of posterior glenoid wear with greater bone loss provides insight into the higher OL associated with type B glenoid compared to type A glenoid. Collectively, these observations support a mechanistic link in which humeral osteophytes interact with the posterior glenoid, driving progressive retroversion, medialization, and the evolution from concentric to eccentric glenoid morphology.

Misir et al^23^ reported consistently higher glenoid version and dimensional measurements in the dominant shoulder, while another study demonstrated a strong correlation between dominant and nondominant shoulders across measurement parameters. These findings align with results from external athletic populations. Professional baseball pitchers exhibit significantly glenoid retroversion in their dominant shoulders compared to their nondominant side,^8^ a side-to-side difference that is not observed in nonthrowing group or the general population.^13^^,^^14^^,^^41^ Similarly, athlete engaged in repetitive overhead activities consistently show increased external rotation, decreased internal rotation, and greater humeral retroversion in the dominant extremity.^30^^,^^34^ Therefore, relying on the contralateral shoulder as a reference for glenoid dimensions and/or version may lead to inaccurate measurements, incompatible component placement, and a higher risk of post-operative complications.

The present systematic review is strengthened by providing an updated literature review that offers insights for future investigations. However, there are several limitations. The current body of literature generally exhibits low methodological quality and a lack of comparative studies, making direct comparisons between different groups challenging. The use of axillary radiographs in a few studies have inherent limitations in accurately characterizing glenoid version and Walch classification when compared with CT-based measurements. Variability in patient positioning, beam angulation, and scapular orientation may also introduce measurement errors and contribute to heterogeneity in reporting associations, especially for glenoid version and subtype classification. Furthermore, to establish a clear association between age with glenoid dimensions, glenoid version, or glenoid wear patterns, longitudinal studies are necessary to track changes in these factors over the lifespan. These limitations highlight the need for further research using higher-quality methodologies to enable comparative evaluations and the development of comprehensive guidelines.

## Conclusion

This systematic review demonstrates that sex and height are consistently associated with larger glenoid dimensions, whereas associations with glenoid version are variable and influenced by population and measurement heterogeneity. Age-related changes suggest that glenoid morphology evolves over time. Eccentric wear patterns, particularly the B2 glenoid, are strongly associated with advancing GH OA, retroversion, posterior humeral subluxation, and bone loss, highlighting the role as marker for disease progression rather than isolated anatomic variation. These findings underscore the need for individualized surgical planning in TSA. Future large scale, longitudinal studies integrating imaging and molecular data are required to move beyond descriptive associations toward clinically useful prediction of pathologic glenoid remodeling.

## Disclaimers

Funding: The present work was supported by the National Institute of Arthritis and Musculoskeletal and Skin Diseases of the National institute of Health under Award Number R01 AR074989. The views expressed are those of the authors and not necessarily those of the funding agency.

Conflicts of interest: The authors, their immediate families, and any research foundation with which they are affiliated have not received any financial payments or other benefits from any commercial entity related to the subject of this article.
