# Supplementary material for: Demographic and anatomic predictors of glenoid morphology: a systematic review
Source: JSES Rev Rep Tech. 2026 Jan 29;6(2):100676. doi: 10.1016/j.xrrt.2026.100676 (PMC12969669; doi:10.1016/j.xrrt.2026.100676)
Supplement: Supplementary Table S1 [file mmc1.docx]

**Glenoid Morphology Literature Search**

**Key Word Search**

| **#** | **Search Term** | **Scope** | **#** **of Results** |
| --- | --- | --- | --- |
| 1 | exp Shoulder Joint/ab, ah, gd [Abnormalities, Anatomy & Histology, Growth & Development] | Glenohumeral joint  Glenohumeral joints  Glenoid labrum  Joint, glenohumeral  Joint, shoulder  Joints, glenohumeral  Joints, shoulder  Labrum, glenoid  Shoulder joint  Shoulder joints | 1300 |
| 2 | exp Glenoid Cavity/ab, ah, gd [Abnormalities, Anatomy & Histology, Growth & Development] | Cavities, glenoid  Cavity, glenoid  Fossa, glenoid  Fossas, glenoid  Glenoid cavity  Glenoid fossa  Glenoid fossa of the scapula | 68 |
| 3 | exp Osteoarthritis/ | Arthritides, degenerative  Arthritis, degenerative  Arthroses  Arthrosis  Degenerative arthritides  Degenerative arthrisis  Osteoarthritides  Osteoarthritis  Osteoarthroses  Osteoarthrosis  Osteoarthrosis deformans | 75262 |
| 4 | Scapula/ab, ah, gd [Abnormalities, Anatomy & Histology, Growth & Development] | Scapula  Scapulae  Shoulder blade  Shoulder blades | 1058 |
| 5 | Glenoid Morphology.mp. | ------------------------------------------- | 117 |
| 6 | Glenoid Shape.mp. | ------------------------------------------- | 29 |
| 7 | Glenoid Version.mp. | ------------------------------------------- | 240 |
| 8 | Glenoid Development.mp. | ------------------------------------------- | 3 |
| 9 | Glenoid Remodeling.mp. | ------------------------------------------- | 10 |
| 10 | Glenoid Bone Loss.mp. | ------------------------------------------- | 570 |
| 11 | exp Joint Instability/ | Hypermobilities, joint  Hypermobility, joint  Instabilities, joint  Instability, joint  Joint hypermobilities  Joint hypermobility  Joint instabilities  Joint instability  Joint laxities  Joint laxity  Laxities, joint  Laxity, joint | 23044 |
| 12 | Glenoid Erosion.mp. | ------------------------------------------- | 157 |
| 13 | A1 glenoid.mp | ------------------------------------------- | 3 |
| 14 | A2 glenoid.mp | ------------------------------------------- | 3 |
| 15 | B1 glenoid.mp | ------------------------------------------- | 3 |
| 16 | B2 glenoid.mp. | ------------------------------------------- | 35 |
| 17 | C1 glenoid.mp. | ------------------------------------------- | 0 |
| 18 | C2 glenoid.mp. | ------------------------------------------- | 2 |
| 19 | Glenoid Inclination.mp. | ------------------------------------------- | 98 |
| 20 | Glenoid Form.mp. | ------------------------------------------- | 3 |
| 21 | Glenoid.mp. | ------------------------------------------- | 6015 |
| 22 | Glenoid Dimension OR Glenoid Dimensions.mp. | ------------------------------------------- | 13 |
| 23 | Glenoid Defect OR Glenoid Defects.mp. [mp=title, book title, abstract, original title, name of substance word, subject heading word, floating sub-heading word, keyword heading word, organism supplementary concept word, protocol supplementary concept word, rare disease supplementary concept word, unique identifier, synonyms] | ------------------------------------------- | 252 |
| 24 | Glenoid Deformity OR Glenoid Deformities.mp. [mp=title, book title, abstract, original title, name of substance word, subject heading word, floating sub-heading word, keyword heading word, organism supplementary concept word, protocol supplementary concept word, rare disease supplementary concept word, unique identifier, synonyms] | ------------------------------------------- | 54 |
| 25 | Glenoid Reconstruction or Glenoid Reconstructions.mp. [mp=title, book title, abstract, original title, name of substance word, subject heading word, floating sub-heading word, keyword heading word, organism supplementary concept word, protocol supplementary concept word, rare disease supplementary concept word, unique identifier, synonyms] | ------------------------------------------- | 98 |
| 26 | Critical Glenoid Bone Loss.mp. | ------------------------------------------- | 14 |
| 27 | Glenoid Vault.mp. | ------------------------------------------- | 73 |
| 28 | Glenoid Retroversion.mp. | ------------------------------------------- | 190 |
| 29 | Glenoid Replacement.mp. | ------------------------------------------- | 31 |
| 30 | Glenoid Component OR Glenoid Components.mp. [mp=title, book title, abstract, original title, name of substance word, subject heading word, floating sub-heading word, keyword heading word, organism supplementary concept word, protocol supplementary concept word, rare disease supplementary concept word, unique identifier, synonyms] | ------------------------------------------- | 737 |
| 31 | Attritional Glenoid.mp | ------------------------------------------- | 5 |
| 32 | exp Cartilage, Articular/ab, ah, gd [Abnormalities, Anatomy & Histology, Growth & Development] | Articular cartilage  Articular cartilages  Cartilage, articular  Cartilages, articular | 2575 |
| 33 | exp Shoulder/ab, ah, gd [Abnormalities, Anatomy & Histology, Growth & Development] | Shoulder  Shoulders | 721 |
| 34 | Osteoarthritis/et, pa, pp, di [Etiology, Pathology, Physiopathology, Diagnosis] | ------------------------------------------- | 30463 |
| 35 | Exp Rotator Cuff/ab, ah, gd [Abnormalities, Anatomy & Histology, Growth & Development] | Cuff, rotator  Infraspinatus  Rotator cuff  Rotator cuffs  Subscapularis  Supraspinatus  Teres minor | 301 |
| 36 | exp Humeral Head/ab, ah, gd, pa [Abnormalities, Anatomy & Histology, Growth & Development, Pathology] | Humeral head  Humeral heads  Humerus head  Humerus heads | 203 |
| 37 | exp Bone Density/ | Bone densities  Bone density  Bone mineral content  Bone mineral contents  Bone mineral densities  Bone mineral density  Density, bone  Density, bone mineral | 59916 |
| 38 | exp “Range of Motion, Articular”/ | Flexibility, joint  Joint flexibility  Joint range of motion  Passive range of motion  Range of motion  Range of motion, articular | 59319 |
| 39 | Exp Case-Control Studies/ | case base studies  case comparison studies  case control studies  case control study  case referent studies  case referrent studies  case-base studies  case-comparison studies  case-comparison study  case-compeer studies  case-control studies  case-control studies, matched  case-control studies, nested  case-control study  case-control study, matched  case-control study, nested  case-referent studies  case-referent study  case-referrent studies  case-referrent study  matched case control studies  matched case-control studies  matched case-control study  nested case control studies  nested case-control studies  nested case-control study  studies, case control  studies, case-base  studies, case-comparison  studies, case-compeer  studies, case-control  studies, case-referent  studies, case-referrent  studies, matched case-control  studies, nested case-control  study, case control  study, case-comparison  study, case-control  study, case-referent  study, case-referrent  study, matched case-control  study, nested case-control | 1387970 |
| 40 | Exp Risk Factors/ | correlates, health  factor, risk  factor, social risk  factors, social risk  health correlates  population at risk  populations at risk  risk factor  risk factor score  risk factor scores  risk factor, social  risk factors  risk factors, social  risk score  risk scores  score, risk  score, risk factor  social risk factor  social risk factors | 946701 |

**Potential “OR” Statements – Need to Clean Up**

1. Shoulder Joint (1), Glenoid Cavity (2), Scapula (4), Glenoid (22), Glenoid Vault (28), Cartilage (34), Shoulder (35), Humeral Head (38), Bone Density (39), Range of Motion (40
2. Osteoarthritis (3), Glenoid Morphology (5), Glenoid Shape (6), Glenoid Version (7), Glenoid Development (8), Glenoid Remodeling (9), Glenoid Bone Loss (10), Joint Instability (11), Glenoid Erosion (12), A1 Glenoid (13), A2 Glenoid (14), B1 Glenoid (15), B2 Glenoid (16), C1 Glenoid (17), C2 Glenoid (18), Glenoid Inclination (20), Glenoid Form (21), Glenoid Dimensions (23), Glenoid Defects (24), Glenoid Deformity (25) Glenoid Reconstruction (26), Critical Glenoid Bone Loss (27), Glenoid Retroversion (29), Glenoid Replacement (30), Glenoid Component (31), Attritional Glenoid (32), Glenoid Cartilage Defect (33), Osteoarthritis (36)

**Potential Intervention Terms**

1. Case-Control Studies OR Case-Control OR Case-Control Study
2. Cause OR Causes
3. Exposure OR Exposures
4. Risk Factor OR Risk Factors OR Risk OR Risks
5. Age OR Aging OR Adolescent OR Elderly OR Young Adult OR Adult OR Middle Aged OR Child OR “80 and over”
6. Gender OR Male OR Female
7. Occupation
8. Sport OR Sports OR Athlete

**Limiting Terms**

1. Human
2. English
